# Resonant Scanning with Large Field of View Reduces Photobleaching and Enhances Fluorescence Yield in STED Microscopy

**DOI:** 10.1038/srep14766

**Published:** 2015-10-01

**Authors:** Yong Wu, Xundong Wu, Rong Lu, Jin Zhang, Ligia Toro, Enrico Stefani

**Affiliations:** 1 Division of Molecular Medicine, Department of Anesthesiology, David Geffen School of Medicine, University of California, Los Angeles, CA, 90095, USA; 2 Department of Physiology, David Geffen School of Medicine, University of California, Los Angeles, CA, 90095, USA; 3 Department of Molecular & Medical Pharmacology, David Geffen School of Medicine, University of California, Los Angeles, CA, 90095, USA; 4 Cardiovascular Research Laboratory, David Geffen School of Medicine, University of California, Los Angeles, CA, 90095, USA

## Abstract

Photobleaching is a major limitation of superresolution Stimulated Depletion Emission (STED) microscopy. Fast scanning has long been considered an effective means to reduce photobleaching in fluorescence microscopy, but a careful quantitative study of this issue is missing. In this paper, we show that the photobleaching rate in STED microscopy can be slowed down and the fluorescence yield be enhanced by scanning with high speed, enabled by using large field of view in a custom-built resonant-scanning STED microscope. The effect of scanning speed on photobleaching and fluorescence yield is more remarkable at higher levels of depletion laser irradiance, and virtually disappears in conventional confocal microscopy. With ≥6 GW∙cm^−2^ depletion irradiance, we were able to extend the fluorophore survival time of Atto 647N and Abberior STAR 635P by ~80% with 8-fold wider field of view. We confirm that STED Photobleaching is primarily caused by the depletion light acting upon the excited fluorophores. Experimental data agree with a theoretical model. Our results encourage further increasing the linear scanning speed for photobleaching reduction in STED microscopy.

Stimulated Emission Depletion (STED) is a powerful technique in fluorescence microscopy that breaks the classical diffraction limit in a purely physical way. In STED microscopy, superresolution is achieved by adding a depletion laser beam with a doughnut-shaped focal spot, which is overlapped over the excitation focal spot to inhibit the peripheral fluorescence and record fluorescence only from the small central hole^1^. In theory, STED microscopy can reach unlimited resolution by indefinitely increasing the depletion laser irradiance. In practice, however, its resolution and signal-to-noise ratio are limited by various factors, most notably severe photobleaching caused by the powerful depletion laser beam^2^,^3^. Therefore, in order to enhance resolution and to improve image quality for STED microscopy, reducing photobleaching is essential.

STED microscopy needs high depletion laser irradiance to reach high resolution. High excitation laser irradiance is often preferred as well to speed up the imaging process. Photobleaching has a linear dependence on weak excitation light irradiance with one-photon excitation (1PE), but for two-photon excitation (2PE) the dependence could have an order of three or higher^4^,^5^. High excitation irradiance may also introduce nonlinearity even in 1PE^6^. In pulsed STED microscopy, the pulse duration of the depletion laser beam needs to be controlled to avoid high-order photobleaching^2^. The triplet states play a crucial role in photobleaching^7^,^8^. It was discovered that lowering the pulsed laser repetition rate down to <1 MHz greatly diminishes photobleaching of the Atto 532 dye. At low repetition rate, the fluorophores have enough time between consecutive pulses to relax from the triplet states, and fluorescence signal was increased by 5–25-fold^9^. The T-Rex (triplet relaxation) technique uses low repetition-rate excitation to reduce photobleaching in STED microscopy^10^. However, low repetition-rate excitation results in slow imaging speed. T-Rex can bunch laser pulses to somewhat speed up imaging without losing the benefit of triplet relaxation^11^.

Another strategy to reduce triplet-state buildup is to adopt resonant scanning^12^,^13^. For example, resonant scanning was applied to continuous-wave (CW) STED microscopy to reduce excessive photobleaching^14^. Furthermore, resonant scanning can facilitate imaging rapid changes in living organisms^15^,^16^. Photobleaching reduction due to fast scanning is nontrivial. A faster scanning speed results in shorter exposure time per scan, but the total exposure time can be kept unchanged by accumulating more scans to reach the same, if not higher, level of fluorescence yield^12^. As previously reported, we built a STED microscope based on an 8 KHz resonant scanner that could reach ~40 nm resolution in a large 50 × 50 μm field of view (FOV)^17^. Because the frequency of the resonant scanning mirror is fixed, a wider FOV would result in a faster scanning speed. We further expanded this microscope by adding a second channel and an ultrafast photon counting acquisition system^18^. In this paper, we use our custom-built STED microscope as a platform to quantitatively investigate the relationship between the linear scanning speed and the photobleaching rate. Although fast scanning is a conventional method to reduce photobleaching, as far as we know there is no careful study of this issue. Clarification of this relation is required to determine whether further increasing scanning speed is worthwhile.

## Theory

### Photobleaching measurement at different scanning speeds maintaining the same illumination dose

When comparing the photobleaching rate at different scanning speeds, we must maintain the same illumination dose in a unit area. In laser scanning confocal microscopy, image acquisition involves: 1) horizontal (*x* axis) scanning the sample with laser irradiance *I* and a linear speed *v*; 2) repetition of the horizontal scan for *L* sequential lines in the vertical direction (*y* axis) with a constant spacing Δ*y* to generate a frame; and 3) the generation of the final image by summing over *F* frames. To maintain the same illumination dose in a unit area, we must have


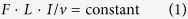


With two different scanning speeds *v*_1_ and *v*_2_, there are two ways to satisfy Eq. (1): 1) maintaining the same laser irradiance (*I*_1_ = *I*_2_ and 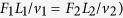, which we will call the equal-irradiance condition; and 2) maintaining the same total number of lines scanned to which we referred as the equal-lines condition, where 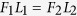 and 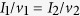.

**Figure 1:**
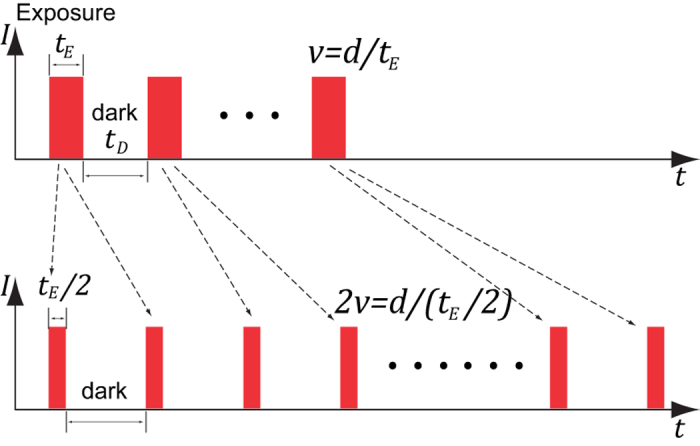
Irradiance received by a fluorophore as a function of time under the equal-irradiance condition. At a linear scanning speed *v*, a fluorophore is exposed to illumination during the exposure time-span 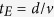 (*d* is the focal spot diameter). When the speed doubles, the exposure time-span is reduced to 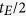. To maintain the same illumination dose, the number of scans needs to be doubled.

We will mainly discuss the equal-irradiance condition to avoid the complication of nonlinearity^6^. Figure 1 shows light exposure under the equal-irradiance condition with a fast and a slow scanning speed. The plots illustrate the irradiance received by fluorophores as a function of time. At speed *v*, in each line the fluorophore is exposed to light during a time-span (we call it exposure time-span) 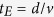, where *d* is the diameter of the laser focal spot (for simplicity we approximate the focal spot with a uniform circular disk). When the scanning speed is doubled, the same dose of illumination is split into two exposure time-spans, each of which has a duration of 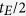. The two exposure time-spans are separated by another time-span without illumination, which we call the dark time-span *t*_*D*_. In general, if the scanning speed is increased by a factor of *n*, the exposure time is shortened to 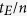. We call *n* the exposure divisor. To maintain the same illumination dose, when the scanning speed is increased by *n* times, the number of scans has to be increased by the same factor.

### Electronic states of a typical organic fluorophore: an analytic model

**Figure 2:**
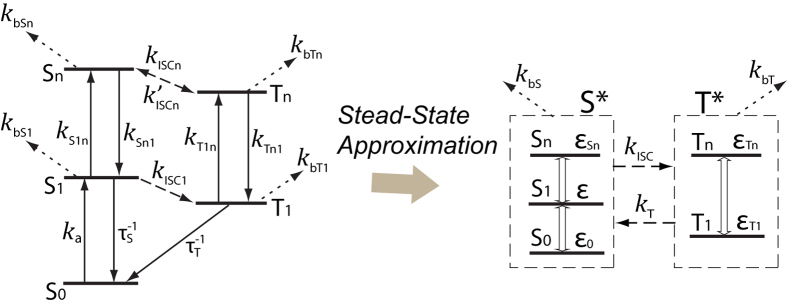
Electronic states of a typical organic fluorophore. The ground state *S*_*0*_ goes to the first excited state *S*_*1*_ via light absorption. With a lifetime of τ_*s*_, relaxation from *S*_*1*_ could produce fluorescence. *S*_*1*_ could also undergo intersystem crossing (ISC) and enter the first triplet state *T*_*1*_, which has a lifetime 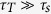. Further excitations from *S*_*1*_ and *T*_*1*_ reach *S*_*n*_ and *T*_*n*_, respectively, which are connected by ISC and reverse ISC. Photobleaching could happen from all excited states. With the steady-state approximation, system reduced to 2 mixture-states S^*^ (all singlet states) and T ^*^ (all triplet states). Within S^*^ and *T*^ *^, the transitions are fast and the relative populations of states (*ε*_0_, *ε*, and *ε*_*n*_, in S^*^; *ε*_*T*1_ and *ε*_*Tn*_ in *T*^ *^) are considered constant over time.

The electronic states of a fluorophore can be modeled by a 5-state system, as depicted in Fig. 2^6^,^9^. We use S^*^ to represent the collection of all singlet states, and T ^*^ to represent all triplet states. Since the transitions within S^*^ or T ^*^ are much faster than the transitions between S^*^ and *T*^ *^ (intersystem crossing and triplet-state relaxation) and the rates of photobleaching (*k*_*bS*_ and *k*_*bT*_), we can apply the steady-state approximation^6^ where the relative populations of the inner states within S^*^ and T ^*^ can be considered constant over time. We quantify photobleaching by the survival probability, which is the probability that a fluorophore could survive from photobleaching and maintain the capacity of emitting fluorescence. With the stead-state approximation, we analytically solved the time dependence of the survival probability 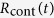 of fluorophores under quasi-continuous laser illumination (CW or high-repetition-rate pulsed lasers) (see Supplementary Information for deduction)


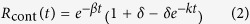


where *β* is related to the rate of photobleaching and *k* to the transition rates between S^*^ and T^*^, and *δ* is roughly the ratio between the triplet-state photobleaching rate and the intersystem transition rates. Since photobleaching is much slower than all other transitions, we have 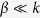, and 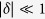. These parameters are all derived from the rate constants in Fig. 2 (see Supplementary Information). Note that the rate “constants” may actually be dependent on the laser irradiance, possibly in a nonlinear manner^6^.

Eq. (2) is for quasi-continuous illumination. With resonant scanning, illumination is split into shorter exposure time-spans (see Fig. 1). When a single short exposure time-span *t*_*E*_ is followed by a much longer dark time-span *t*_*D*_ which is also much longer than the triplet-state lifetime, and when photobleaching from the higher energy triplet state *T*_*n*_ dominates over that from *T*_*1*_^12^,^19^, the fluorophore survival probability after scanning for a time 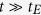 is


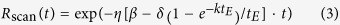


where 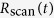 decays exponentially with a time constant of 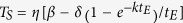, and 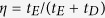 is the scanning duty cycle. We call the time constant *T*_*s*_ the fluorophore survival time^20^.

In a single frame, a fluorophore is scanned for ~*d*/Δ*y* vertical lines. Recall that *d* is the focal-spot diameter, and Δ*y* is the pixel height. In our experiments, 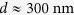 and Δ*y* = 15 nm, and therefore a fluorophore is scanned for ~20 times in each frame. With an 8 KHz scanning mirror and bidirectional scanning, each cycle is 62.5 μs, and the dark time-span lasts for ~60 μs. If the triplet-state lifetime is much shorter than 60 μs (e.g., the Atto 532 dye has a triplet-state lifetime of ~1 μs^9^), then the population of the triplet states vanishes for each line.

Many fluorescent dyes could stay in the triplet states for milliseconds or longer. For example, Atto 647N was reported to have a triplet-state lifetime of ~27 ms^21^,^22^ with oxygen removal. Oregon Green 488 stays in the triplet state for milliseconds when mounted in polyvinyl alcohol^23^. For these dyes, we consider each frame as one cycle. The dark time-span then depends on the acquisition frame rate, which is determined by the number of lines per frame. If each frame has 1,000 lines, the dark time-span for each fluorophore is ~62.5 ms, which is long enough for triplet relaxation. Considering the rounded shape of the focal spot, the exposure time-span is approximated by 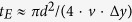. Illumination within the exposure time-span can be considered quasi-continuous and Eq. (2) and Eq. (3) still hold.

Under the equal-irradiance condition, an *n*-fold increase in scanning speed divides *t*_*E*_ into *n* equal portions, where *n* is the exposure divisor. After *t*_*E*_, the fluorescence gain ratio 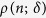, defined as the ratio of the remaining fluorescence between the *n*-fold faster speed and the original speed, is


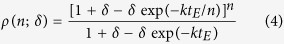


**Figure 3:**
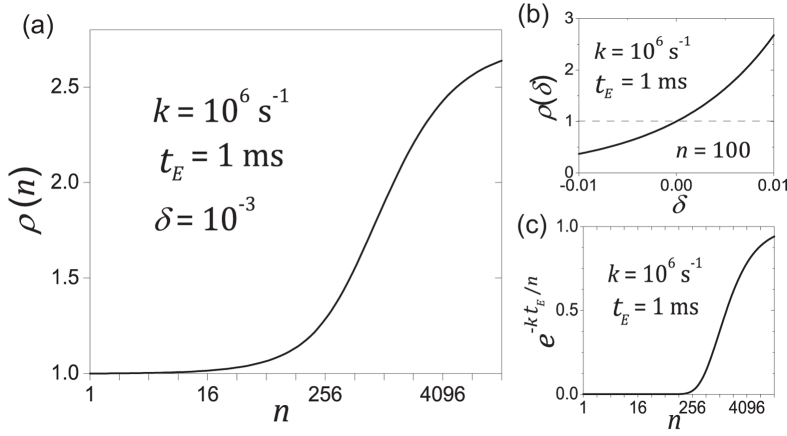
Typical profile of *ρ*(*n*; *δ*), the ratio of fluorescence gain through splitting the exposure time-span *t*_*E*_ into *n* pieces, as described by Eq. (4). (**a**) Profile of 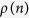 for a given *δ*. (**b**) For a given *n*, 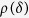 is a monotonically increasing function. (**c**) Profile of 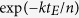, which determines the profile of 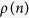. The chosen values of the parameters are based on experiments (see Fig. 7 and Table 2).

This ratio is dependent on the parameters *k* and *δ.* The parameter *k* quantifies the rate of the triplet-state dynamics (including intersystem crossing and triplet-state relaxation), and the parameter *δ* signifies the rate of photobleaching via the triplet-state pathway. Some features of 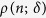 are: 1) fast scanning slows down photobleaching (i.e., 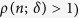 only when *δ* > 0, or equivalently, 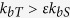 (see Supplementary Information). Intuitively, this is when the triplet-state photobleaching dominate over the singlet-state photobleaching pathway; 2) 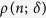 is a monotonically increasing function of *δ* (Fig. 3b). Therefore, the effect of the scanning speed is more remarkable with growing *δ* > 0; 3) for a given positive *δ*, the profile of 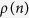 is determined by 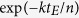. Figure 3a illustrates the profile of 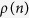, and Fig. 3c shows the profile of 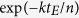. The two profiles are very similar. The slow entry of 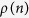 corresponds to the very small values of 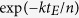, the saturation happens when 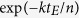 approaches one, and in between is a quasi-linearly growing phase; and 4) at the limit of 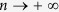, the fluorescence gain ratio approaches approximately 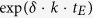, and the remaining fluorescence after *t*_*E*_ approaches 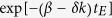.

## Experimental Methods

The experiments were conducted using our custom-built resonant-scanning dual-channel STED microscope with an ultrafast photon counting system^17^,^18^. In brief, fluorescence excited by two pulsed/CW dual-mode lasers, with wavelengths of 635 nm and 488 nm (LDH-D-C-635 and LDH-D-C-485, PicoQuant, Germany), are depleted by a 750 nm pulse laser (Mai Tai HP, Newport, USA) and by a 592 nm CW fiber laser (2RU-VFL-P-2000-592, MPB Communications, Canada), respectively. To ensure excellent linearity of the acquisition system, photomultipliers (H7422-40, Hamamatsu Photonics, Japan) were used as detectors whenever possible (with piezo-stage scanning, an avalanche-photodiode photon-counting module was used. See below). The resonant scanner (CRS 8 KHz, Cambridge Technology, USA) has a fixed frequency and we control the scanning speed by changing the excursion of the horizontal resonant scanning mirror that determines the width of the field of view (FOV). The movement of the resonant scanner is sinusoidal but is almost linear at the image center. We thus cropped the image around the center where the scanning speed was no less than 90% of the maximum speed 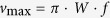, where *W* is the full FOV width, and *f* = 8 KHz is the frequency of the resonant scanner.

Table 1 lists the information of three different zooms (1, 4 and 8), which is defined by the width of the FOV, and the corresponding linear scanning speeds. A visualization of these zooms is shown in Supplementary Fig. S1. We kept the same pixel size (15 × 15 nm) and the same number of lines in a frame for all zooms. As discussed in the previous section, to satisfy the equal-irradiance condition, the total number of frames taken at Zoom 1 must be 8-fold as many as at Zoom 8, which would result in 8-fold longer imaging time.

We measured the photobleaching rate by taking a time series of images for the same FOV and recording the decay of image intensity (after subtracting a predetermined background caused by parasite light and dark counts). For each specimen, the fluorescence signal could vary greatly in different regions. Therefore, for each FOV, we normalized the image intensity to the first image. Each data point includes the mean value plus the standard error calculated from at least 4 series of images in different regions. The illumination dose is measured by the normalized imaging time, which is defined by the actual imaging time to reach a certain illumination dose at Zoom 4 with predetermined laser irradiance. Therefore, by definition two experiments complete in equal normalized imaging times must receive the same dose of illumination. For example, under the equal-irradiance condition, a normalized imaging time of 10 seconds would mean 40 seconds of actual imaging at Zoom 1, or 5 seconds of actual imaging at Zoom 8.

Varying the zoom from 1 to 8 by changing the FOV width with resonant scanning provided up to 8-fold change in linear scanning speed. To study the change in a wider range, we also employed a piezoelectric stage system (Nano PDQ 3-axis, controlled by Nano-Drive 85, 20 bit, Mad City Labs, USA) to access very slow scanning speed. With the piezo-stage, we control the pixel size and the pixel dwell time to determine the scanning speed. We kept the pixel dwell time at 1 ms in all experiments. Different pixel sizes were chosen to satisfy the equal-irradiance condition and the equal-lines condition, respectively (see Table 1). When the piezo-stage was used or compared to, an avalanche photodiode single photon counting module (SPCM-AQRH-12, Perkin Elmer, USA) was employed to detect fluorescence. Neutral density filters were used to when fluorescence was too strong to maintain detection linearity.

We studied six common STED dyes: Atto 647N (Atto Tech, Germany), Oregon green 488 (Life Technologies, USA), Abberior STAR 635P (Abberior, Germany), Chromeo 494 (Active Motif, USA) , Alexa Fluor 647, and Alxea Fluor 488 (Life Technologies, USA). The information of the dyes and the samples is listed in Supplementary Table S1. All specimens were mounted with ProLong® Gold (Life Technologies, USA) (see Supplementary Information for the protocol).

We used the ImageJ (NIH, USA) and the Origin 7.5 (Origin Lab, USA) software applications to visualize and analyze the data.

## Results

### Fast scanning significantly slows down photobleaching when depletion irradiance is high

We detected the fluorescence decay of Atto 647N in Sample #1 as a function of illumination dose with varying depletion laser irradiance. During the experiments, the 635 nm excitation laser beam had 0.22 mW optical power (throughout this paper, the laser power was always measured at the back aperture of the objective and the values are time-averages), or ~800 kW∙cm^−2^ irradiance (time-average) at the focal spot. The depletion laser had up to 110 mW power, or up to ~12 GW∙cm^−2^ peak irradiance in focus. These values are what we would use in practical STED imaging experiments, in which we need to set the excitation power high for fast imaging speed and the depletion power high for optimal resolution.

**Figure 4:**
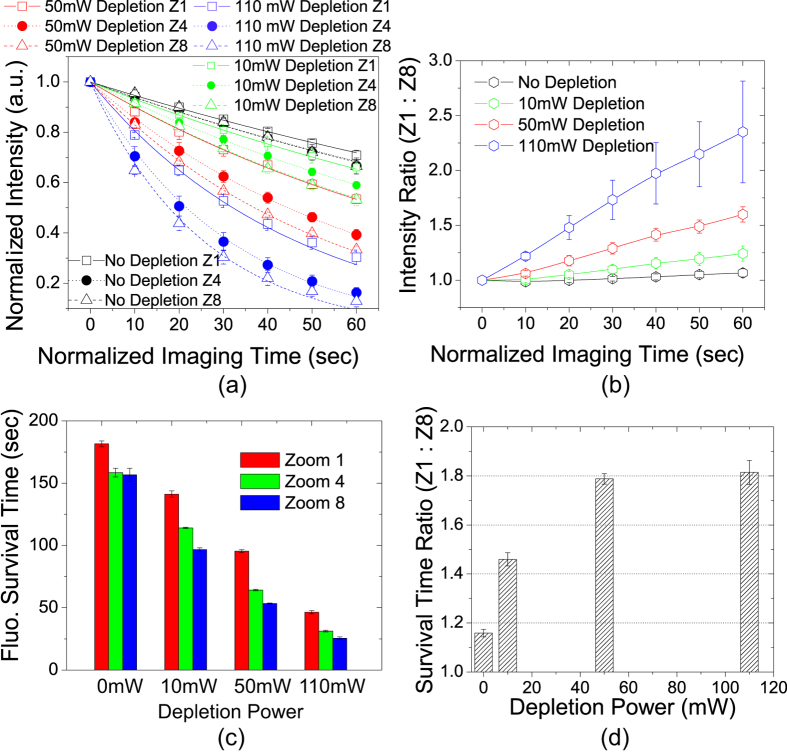
Photobleaching rates in Sample #1 with varying depletion laser power. (**a**) Normalized image intensity decays as a function of illumination dose measured by the normalized imaging time. Data points were fitted to mono-exponential decay expressed in Eq. (3). (**b**) Intensity ratio between Zoom 1 and Zoom 8 is always greater than one, and increases with time and growing depletion laser power. Lines are a guide for the eye. (**c**) Fluorophore survival time with different depletion laser power at different zooms. Lower zooms (faster scanning speed) have longer survival time. (**d**) Ratio of the survival time between Zoom 1 and Zoom 8 is greater than one and increases with growing depletion power.

Figure 4a shows the normalized image intensity decay as a function of the normalized imaging time in Sample #1. The lower zooms (faster scanning speeds) resulted in slower decay: the decay rate with 50 mW depletion power at Zoom 1 was almost the same as that with 10 mW depletion power at Zoom 8. This means widening the FOV by a factor of 8 would allow us to increase the depletion power by 5 times, which would reach much higher resolution, without causing more photobleaching. Figure 4b shows the ratio of the remaining fluorescence signal between Zoom 1 and Zoom 8 as a function of illumination dose. The ratio is always greater than one and increases with higher depletion laser power, demonstrating that the advantage of faster scanning becomes more significant at higher depletion laser irradiance. With 110 mW depletion power, the remaining fluorescence at Zoom 1 is up to ~235 ± 46% as high as at Zoom 8 in 1 minute of normalized imaging time. In Fig. 4a, the data points were fitted to a mono-exponential decay function as expressed in Eq. (3), and the survival time (normalized) values are displayed in Fig. 4c. To quantify the difference in photobleaching rates at different zooms, the ratio of the survival time between Zoom 1 and Zoom 8 is plotted as a function of the depletion laser power in Fig. 4d. The ratio is always greater than one and increases with growing depletion laser power. With 50 mW depletion power (~6 GW∙cm^−2^ peak irradiance), the survival time at Zoom 1 is 95 ± 1 seconds, while at Zoom 8 is 53 ± 0.4 seconds. The ratio is thus ~1.79 ± 0.02. In other words, 8-fold higher scanning speed (as a result of 8-fold wider FOV) can slow down the rate of photobleaching by ~80%.

The same experiment was repeated for Sample #2 and the results are shown in Supplementary Fig. S2. Sample #2 is a rat heart tissue section, which is much brighter and bleaches much slower than Sample #1. The image intensity decay curves of this sample often do not fit well to mono-exponential decay, probably due to its higher fluorophore concentration^24^ or more complex molecular environment. In spite of these differences, the relation between the scanning speed and the photobleaching rate in Sample #2 is qualitatively the same as in Sample #1. For example, with 110 mW depletion power, Zoom 1 yielded up to ~80% more fluorescence than Zoom 8 in 1 minute of normalized imaging time (see Supplementary Fig. S2b).

**Figure 5:**
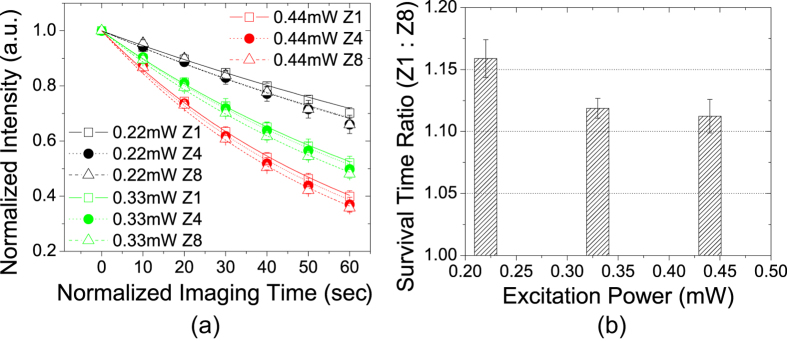
Photobleaching in Sample #1 in regular confocal microscopy (zero depletion). (**a**) Image intensity decay curves fitted to Eq. (3). (**b**) Ratio of survival time between Zoom 1 and Zoom 8 as a function of excitation power. Fluorophore survival time is similar at all 3 zooms. Difference is at most ~15% with the lowest excitation power. At higher excitation power, the difference further diminishes.

As shown in Fig. 4d, the effect of photobleaching slowing down by fast scanning is least notable when the depletion laser power is zero, i.e., for regular confocal microscopy. To confirm the effect was indeed related to depletion rather than a universal property only related to the photobleaching rate itself, we increased the excitation laser power in regular confocal condition and repeated the experiment. The results for Sample #1 are shown in Fig. 5. When the excitation laser power was increased to 0.44 mW (red curves in Fig. 5a), the photobleaching rates are comparable to 0.22 mW excitation plus 50 mW depletion power at Zoom 8 (red dash curve in Fig. 4a). However, without depletion the effect of scanning speed on photobleaching rate is quite small. The decay curves were fitted to Eq. (3) and the ratio of the survival time between Zoom 1 and Zoom 8 are shown in Fig. 5b. The difference between two zooms is always less than 20%. Furthermore, with higher excitation power (hence faster bleaching rate), the ratio decreases. We also conducted the same experiment for Sample #2 (see Supplementary Fig. S3) and obtained the same result: the scanning speed has little impact on photobleaching rate when depletion is not present.

In summary, the reduction of photobleaching by increasing the scanning speed is very pronounced when the depletion lasers power is high and it is minimal when using only the excitation laser (i.e., conventional confocal microscopy).

### STED photobleaching is primarily due to the depletion laser acting on the excited states

**Figure 6:**
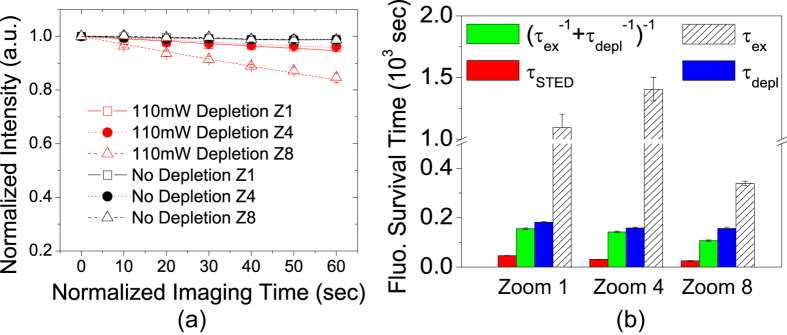
STED photobleaching is primarily caused by depletion light acting on excited states. (**a**) Image intensity decay caused by depletion alone in Sample #1. Red curves are fitting to mono-exponential decay. Black curves represent background photobleaching caused by a low power (16 μW) excitation laser beam, which do not decay. (**b**) Comparison of STED survival time 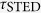, excitation-only survival time 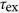, depletion-only time constant 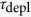, and 
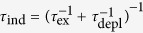
 (would-be survival time if two lasers caused photobleaching independently). One can see that 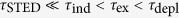.

It was previously demonstrated that, when the duration of 760 nm depletion laser pulses is relatively long (~160 ps), they cause little photobleaching for the RH-414 dye^2^. Our pulsed depletion laser is at 750 nm with pulse duration of ~400 ps, and we tested if the pulses would bleach the Atto 647N dye by themselves. The red curves in Fig. 6a show the fluorescence decay caused by 110 mW depletion in Sample #1. The images were obtained with low power excitation laser beam (16 μW), but the excitation beam and the depletion beam were never simultaneously switched on. To measure the background photobleaching caused by the 16 μW excitation beam, the experiment was repeated with the depletion beam being blocked all the time and the results are shown by the black curves in Fig. 6a. The black curves do not show decaying, and therefore, the red decay curves were caused by the 110 mW depletion laser beam alone. In STED microscopy, if the depletion laser and the excitation laser caused photobleaching independently, one would expect its survival time to be 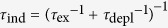, where 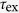 and 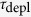 is the survival time of the excitation-only bleaching and the depletion-only bleaching, respectively. From the black curves in Fig. 4a, the blue curves in Fig. 4a, and the red curves in Fig. 6a, the values of 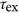, 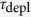, and the real STED survival time 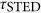 were extracted and plotted in Fig. 6b. It is obvious that 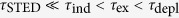. Therefore, we can conclude that the depletion laser must act on the fluorophores in the excited states to account for the photobleaching rates observed in STED microscopy. The same experimental procedure was repeated for Sample #2, and we obtained the same results as shown in Supplementary Fig. S4.

### STED photobleaching cannot be reduced by simply lowering laser power

As discussed in previous sections, there are essentially two ways to distribute a certain dose of illumination to a given FOV, the equal-irradiance condition and the equal-lines condition. Under the equal-lines condition, Zoom 8 should use 1/8 of the excitation irradiance as Zoom 1 uses. Due to two-step photolysis at higher excitation irradiance^6^,^25^, photobleaching has a super-linear (faster than linear) dependence on the excitation laser irradiance. Therefore, lower excitation irradiance is beneficial to slow down photobleaching in confocal microscopy (see Supplementary Fig. S5a). However, STED microscopy has a different story. Because the optical resolution of STED microscopy is determined by the depletion irradiance, one has to maintain the same depletion laser power for all zooms. Therefore, under the equal-lines condition, the depletion illumination dose at Zoom 8 would be 8-fold as high as Zoom 1, which causes more overall photobleaching (see Supplementary Fig. S5b). Therefore, faster scanning speed is still beneficial under the equal-lines condition.

In conclusion, faster scanning speed is not useful in conventional resonant scanning confocal microscopy. Instead of increasing the scanning speed by *n* times, one should lower the excitation irradiance by 1/*n*. However, this approach does not apply to STED microscopy, because the depletion irradiance cannot be lowered without compromising the optical resolution.

### CW STED results

We repeated the above experiments for the Oregon green 488 dye in Sample #3, which was excited and depleted by CW lasers, and most of the results are qualitatively the same as for the Atto 647N dye. In Supplementary Fig. S6, one can see that the faster scanning speed resulted in slower photobleaching, and that such effect is emphasized by higher depletion power. With 220 mW depletion power, the survival time at Zoom 1 is 45 ± 0.6 seconds, ~45 ± 2% longer than at Zoom 8 (31 ± 0.4 seconds) (Supplementary Fig. S6d). This effect is less pronounced compared to pulsed STED, likely because in CW STED the effective depletion irradiance is much lower. In fact, 220 mW CW depletion is comparable to ~10 mW pulsed depletion in terms of instantaneous optical power. Supplementary Fig. S7 demonstrates that, this effect does not exist in regular confocal condition, even when the excitation power was so high that the photobleaching rate was comparable to STED. Supplementary Fig. S8 illustrates photobleaching under the equal-lines condition (red curves): in regular confocal condition, the lower zooms had less photobleaching; in STED, it was the other way around. The only major difference is that the 592 nm depletion laser could cause significant photobleaching by its own, and more so at a higher zoom, as shown in Supplementary Fig. S9a. But STED photobleaching is still much quicker than that caused by the excitation light and the depletion light acting separately in time (Supplementary Fig. S9b). It again supports the theory that STED photobleaching is primarily due to the depletion laser affecting the excited fluorophores.

### Piezo-stage scanning results

**Figure 7:**
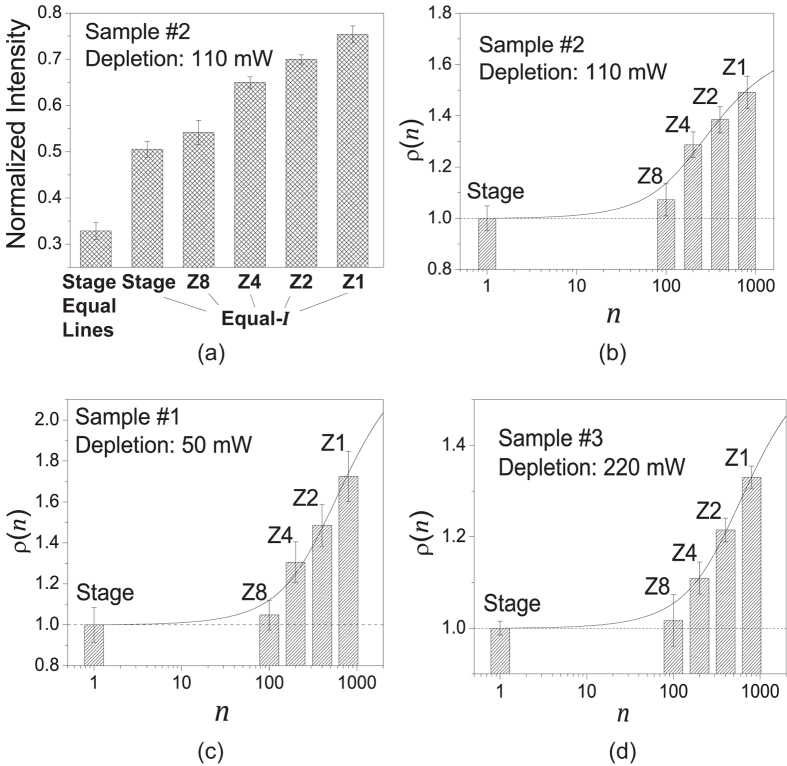
Photobleaching rates of piezo-stage and resonant scanning in STED microscopy. (**a**) Normalized image intensity of the 2nd image in the time series for Sample #2. Under the equal-irradiance condition (pixel size 110 × 100 nm), piezo-state caused about the same photobleaching as resonant scanning at Zoom 8, despite its exposure time-span is 100-fold longer. Under the equal-lines condition (pixel size 15 × 15 nm), piezo-stage resulted in much more severe photobleaching. (**b**–**d**) show fluorescence gain ratio 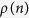 with respect to piezo-stage scanning as a function of exposure divisor *n*, under the equal-irradiance condition for Sample #2, Sample #1 and Sample #3, respectively. Data points (columns) were fitted to 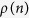 expressed in Eq. (4) (solid lines). Fitted parameters are listed in Table 2.

Scanning speed of a piezo-stage is much slower than a resonant scanner (see Table 1). Under the equal-irradiance condition, Eq. (1) dictates that the pixel size of the piezo-stage ought to be 110 × 110 nm. This size would be too large for a practical STED imaging experiment. The equal-lines condition is what a practical STED imaging experiment would use, under which the pixel size was chosen to be the same as in resonant scanning (15 × 15 nm), the excitation laser power was reduced to 3.9 μW, and the depletion power was kept constant to preserve the optical resolution. In Fig. 7, we show the photobleaching comparison of piezo-stage and resonant scanning. Due to the slowness of piezo-stage, we only took two images for each time series to measure photobleaching (each image with 3 minutes of normalized imaging time). Figure 7a shows the normalized image intensity of the second image to quantify photobleaching in Sample #2. Under the equal-irradiance condition, though the exposure time of the piezo-stage is 100-fold longer than resonant scanning at Zoom 8, their photobleaching rates are about the same. Piezo-stage scanning under the more practical equal-lines condition caused much more severe photobleaching due to the excessive depletion illumination dose. For the four equal-irradiance cases, using the piezo-stage scanning case as the reference, the fluorescence gain 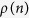 as a function of exposure divisor *n* was plotted in Fig. 7b. Its profile is consistent with the function profile depicted in Fig. 3a. Piezo-stage scanning and Zoom 8 together show the slow entry, while Zoom 4, Zoom 2 and Zoom 1 roughly belong to the quasi-linearly growth phase of the 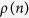 profile. Similar results were obtained for Sample #1 and Sample #3, as illustrated in Fig. 7c,d, respectively. The experimental data points were fitted to the theoretical model expressed by Eq. (4), and the fitted parameter (*k* and *δ*) values are summarized in Table 2.

### Other fluorescent dyes

The experiment results of the other four fluorescent dyes, Abberior STAR 635P, Alexa Fluor 647, Chromeo 494, and Alexa Fluor 488, are summarized in Table 2 and Supplementary Fig. S10. Except Alexa Fluor 647, they all show significant reduction in the photobleaching rate when the depletion power is high. The exception of Alexa Fluor 647 is consistent with a previous study indicating that photobleaching of Alexa Fluor 647 does not involve triplet state^26^. For all the four dyes, the STED fluorophore survival time 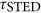 is much shorter than 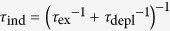, the survival time if the depletion light and the excitation light causing photobleaching independently. Therefore, for all these dyes, the depletion light causes photobleaching primarily by acting on the excited states. For Abberior STAR 635P and Chromeo 494, the depletion light alone does not cause photobleaching at all. Finally, the experimental data of the fluorescence gain ratio 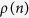 are fitted to Eq. (4), and the fitted parameters are summarized in Table 2.

An interesting observation is that, for Abberior STAR 635P and Chromeo 494, the STED photobleaching rate at Zoom 1 could be slower than the confocal photobleaching rate (Supplementary Fig. S10a1 and S10c1). This is because: 1) in the experiments we used high excitation power, which cause rapid nonlinear photobleaching in confocal microscopy; and 2) when the depletion light is applied, the excited states population diminishes and photobleaching through the singlet-state pathway is suppressed. If this effect wins over the triplet-state photobleaching caused by the high-power depletion light, STED photobleaching will be overall slower than confocal photobleaching.

## Summary and Discussion

The main conclusion of this paper is that the linear scanning speed has a nontrivial impact on the photobleaching rate and fluorescence yield in resonant scanning STED microscopy. Since the frequency of the resonant scanner is fixed, the linear scanning speed can be readily controlled by changing the width of the scan FOV. For five out of six fluorescent dyes we studied, we have shown that an 8-fold wider FOV can extend the fluorophore survival time by 35–80% at high depletion irradiance. When the depletion power is low, this impact diminishes. For regular confocal microscopy, it may disappear altogether. Photobleaching in STED microscopy is more severe than that caused by separate illumination of the depletion laser and the excitation laser, suggesting that the primary mechanism of STED photobleaching requires the fluorophores first being excited by the excitation laser.

Higher depletion irradiance emphasizes the effect of scanning speed on photobleaching, because it induces more complete fluorescence suppression. As discussed in the *Theory* section, 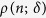 increases with growing *δ* > 0. With higher depletion irradiance, fluorescence suppression is higher and thus the population of the excited states *ε* decreases 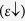. For fluorescent dyes with long triplet-state lifetime, 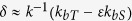 and thus 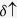. Therefore, 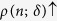 and the effect of scanning speed becomes more notable. On the other hand, when there is no depletion and the excitation power increases, 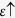 causes 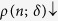, and the effect diminishes.

All the experiments discussed above were designed to resemble practical STED imaging and thus the depletion beam had a doughnut-shaped cross-section. But the zero-intensity center complicates the photobleaching process. We repeated one of the experiments (110 mW depletion power, Sample #2) with a Gaussian depletion beam and compared the result to standard STED in Supplementary Fig. S11. The image intensity curves under two conditions only have minor differences, suggesting that the complexity induced by the hollow center is insignificant.

In STED microscopy, the depletion laser power is of particular importance as it determines the optical resolution. Therefore, we have concentrated on studying the impact of the depletion power on photobleaching. We expect the impact of the excitation power on STED photobleaching to be less important. We illustrate this point by Supplementary Fig. S12, in which we show that the ratio of the fluorophore survival time between Zoom 1 and Zoom 8 remains roughly a constant with three different excitation power for Atto 647N.

We have shown that resonant scanning is far more superior than piezo-stage scanning (with 1 ms pixel dwell time) in terms of photobleaching reduction, mainly because fluorophores in piezo-stage scanning receive excessive depletion illumination (it would have to use the equal-lines condition to reach a pixel size of 15 × 15 nm). For most of the dyes under this study, piezo-stage scanning under the equal-irradiance condition has roughly the same photobleaching rate as at Zoom 8. It indicates that Zoom 8 just enters the quasi-linearly growth phase of Eq. (4). The fitted curves and parameters presented in Table 2 can be used to predict how much potential there is to further increase the scanning speed for slowing down photobleaching. For example, from the solid curve in Fig. 7c (0.22 mW excitation, 50 mW depletion, Sample #1), we can estimate that doubling the scanning speed at Zoom 1 would gain 18% more fluorescence. The maximum gain from keeping increasing the scanning speed is 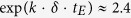, i.e., one can attain up to 140% more fluorescence signal compared to slow scanning.

Resonant scanning STED microscopy with high-repetition-rate pulsed lasers is similar to the bunched T-Rex technique^11^, in the sense that a fluorophore would encounter the same temporal pattern of illumination: illumination bunches in bunched T-Rex are equivalent to exposure time-spans in resonant scanning. However, with resonant scanning one fluorophore’s dark time is another fluorophore’s exposure time, whereas in bunched T-Rex the dark time is universal for all fluorophores. Therefore, resonant scanning is more efficient in time. This is particularly important for the dyes with triplet-state lifetime in milliseconds, for which T-Rex imaging speed would be too slow.

## Additional Information

**How to cite this article**: Wu, Y. *et al.* Resonant Scanning with Large Field of View Reduces Photobleaching and Enhances Fluorescence Yield in STED Microscopy. *Sci. Rep.* **5**, 14766; doi: 10.1038/srep14766 (2015).

## Supplementary Material

Supplementary Information

## Figures and Tables

**Table 1:** Scanning conditions used in experiments.

	FOV size (μm)^a^	Pixel size (nm)	Linear scanning speed (m∙s^−1^)	Exposure time per scan *T*_*E*_ (μs)
Zoom 1	29.6 × 14.4	15 × 15	1.38	3.4
Zoom 4	7.4 × 14.4	15 × 15	0.345	13.7
Zoom 8	3.7 × 14.4	15 × 15	0.173	27.3
Pizeo-stage equal-irradiance	Any	110 × 110	1.1 × 10^−4^	2,730
Pizeo-stage equal-lines	Any	15 × 15	1.5 × 10^−5^	20,000

^a^FOV with ≥90% of the maximum scanning speed.

**Table 2:** Summary of experimental results.

Sample	Dye	Laser power (mW)		Fluorophore survival time (seconds)			Model parameters	
		Ex.	Depl.	Zoom 1	Zoom 8	Ratio (Z1:Z8)	*k*(μs^−1^)	*δ* (10^−3^)
#1	Atto 647N	0.22	50	95(±1)	53(±0.4)	1.79(±0.02)	0.27(±0.1)	1.2(±0.2)
#2	Atto 647N	0.22	110	N.A.			0.14(±0.04)	1.3(±0.2)
#3	Oregon green 488	0.01	220	45(±0.6)	31(±0.4)	1.45(±0.02)	0.32(±0.05)	0.54(±0.04)
#4	Abberior STAR 635P	0.22	110	153(±2)	87(±1)	1.75(±0.02)	0.13(±0.04)	1.2(±0.3)
#5	Alexa Fluor 647	0.03	50	47(±0.6)	45(±0.2)	1.05(±0.01)	0.11(±0.08)	0.29(±0.1)
#6	Chromeo 494	0.03	110	120(±1)	86(±1)	1.39(±0.01)	0.24(±0.03)	0.80(±0.06)
#7	Alexa Fluor 488	0.04	220	39(±0.8)	29(±0.3)	1.35(±0.03)	0.059(±0.005)	2.4(±0.15)
