# Supplementary material for: Resonant Scanning with Large Field of View Reduces Photobleaching and Enhances Fluorescence Yield in STED Microscopy
Source: Sci Rep. 2015 Oct 1;5:14766. doi: 10.1038/srep14766 (PMC4589784; doi:10.1038/srep14766)
Supplement: Supplementary Information [file srep14766-s1.pdf]

Resonant Scanning with Large Field of View Reduces Photobleaching and Enhances Fluorescence Yield in STED Microscopy

Yong Wu, Xundong Wu, Rong Lu, Jin Zhang, Ligia Toro and Enrico Stefani

Supplementary Information

Deduction of Equations

With the steady-state approximation, we only need to consider a 2-state system consisting of S^* and T^*, whose populations P_S and P_T satisfy

$$\begin{cases} \dfrac{dP_S}{dt} = -\left(\varepsilon k_{ISC1} + \varepsilon_{Sn} k_{ISCn} + \varepsilon k_{bS1} + \varepsilon_{Sn} k_{bSn}\right) P_S + \left(\varepsilon_{T1} \tau_T^{-1} + \varepsilon_{Tn} k'_{ISCn}\right) P_T \\ \dfrac{dP_T}{dt} = \left(\varepsilon k_{ISC1} + \varepsilon_{Sn} k_{ISCn}\right) P_S - \left(\varepsilon_{T1} \tau_T^{-1} + \varepsilon_{Tn} k'_{ISCn} + \varepsilon_{T1} k_{bT1} + \varepsilon_{Tn} k_{bTn}\right) P_T \end{cases}$$

where the parameters (the rate constants and the relative populations) are given in Fig. 2. To simplify the equation, we let

$$\begin{aligned} k_{ISC} &= k_{ISC1} + \left(\varepsilon_{Sn} / \varepsilon\right) k_{ISCn} \\ k_{bS} &= k_{bS1} + \left(\varepsilon_{Sn} / \varepsilon\right) k_{bSn} \\ k_T &= \varepsilon_{T1} \tau_T^{-1} + \varepsilon_{Tn} k'_{ISCn} \\ k_{bT} &= \varepsilon_{T1} k_{bT1} + \varepsilon_{Tn} k_{bTn} \end{aligned}$$

where the relative populations are

$$\begin{aligned} \varepsilon &= \frac{k_a k_{Sn1}}{k_a k_{Sn1} + k_a k_{S1n} + \tau_S^{-1} k_{Sn1}} \\ \varepsilon_{Sn} &= \frac{k_a k_{S1n}}{k_a k_{Sn1} + k_a k_{S1n} + \tau_S^{-1} k_{Sn1}} \\ \varepsilon_{T1} &= \frac{k_{Tn1}}{k_{Tn1} + k_{T1n}} \\ \varepsilon_{Tn} &= 1 - \varepsilon_{T1} = \frac{k_{T1n}}{k_{Tn1} + k_{T1n}} \end{aligned}$$

We than have

$$\begin{cases} \dfrac{dP_S}{dt} = -\varepsilon\left(k_{ISC} + k_{bS}\right) P_S + k_T P_T \\ \dfrac{dP_T}{dt} = \varepsilon k_{ISC} P_S - \left(k_T + k_{bT}\right) P_T \end{cases}$$

Since photobleaching is much slower than transitions among the electronic states, we have the first order approximation of the eigenvalues

$$\begin{cases} -\lambda_1 = -\left(\varepsilon k_{ISC} + k_T\right) - \dfrac{\varepsilon^2 k_{bS} k_{ISC} + k_{bT} k_T}{\varepsilon k_{ISC} + k_T} \\ -\lambda_2 = -\dfrac{\varepsilon k_{bS} k_T + \varepsilon k_{bT} k_{ISC}}{\varepsilon k_{ISC} + k_T} \end{cases}$$

and the solution with the initial conditions $P_S(0)=P_0$ and $P_T(0)=0$ is

$$\begin{cases} P_S(t)=P_0\dfrac{1}{\lambda_1-\lambda_2}\left[\left(k_T+k_{bT}-\lambda_2\right)e^{-\lambda_2 t}-\left(k_T+k_{bT}-\lambda_1\right)e^{-\lambda_1 t}\right] \\ P_T(t)=P_0\dfrac{\varepsilon k_{ISC}}{\lambda_1-\lambda_2}\left(e^{-\lambda_2 t}-e^{-\lambda_1 t}\right) \end{cases}$$

Let

$$\alpha=\lambda_1-\left(\varepsilon k_{ISC}+k_T\right)=\frac{\varepsilon^2 k_{bS}k_{ISC}+k_{bT}k_T}{\varepsilon k_{ISC}+k_T}$$

$$\beta=\lambda_2=\frac{\varepsilon k_{bS}k_T+\varepsilon k_{bT}k_{ISC}}{\varepsilon k_{ISC}+k_T}$$

$$k=\lambda_1-\lambda_2=\left(\varepsilon k_{ISC}+k_T\right)+\left(\alpha-\beta\right)$$

and the survival probability of fluorophores under quasi-continuous illumination is

$$R_{\text{cont}}(t)=\left(1+\frac{k_{bT}-\alpha}{k}\right)e^{-\beta t}-\left(\frac{k_{bT}-\alpha}{k}\right)e^{-\alpha t}e^{-\left(\varepsilon k_{ISC}+k_T\right)t}$$

Let

$$\delta=\frac{k_{bT}-\alpha}{k}=\frac{\varepsilon k_{ISC}\left(k_{bT}-\varepsilon k_{bS}\right)}{\left(\varepsilon k_{ISC}+k_T\right)^2}$$

We then have

$$R_{\text{cont}}(t)=e^{-\beta t}\left[\left(1+\delta\right)-\delta e^{-kt}\right]$$

Let the exposure time-span be t_E and the dark time-span be t_D. If, 1) t_D is much longer than the triplet-state lifetime; and 2) photobleaching from T_n dominates over that from T_1 (therefore, photobleaching is negligible during t_D), then the fluorophore survival probability after m scans with imaging time $t=m\left(t_E+t_D\right)$ is

$$R_{\text{scan}}(t)=\left\{e^{-\beta t_E}\left[\left(1+\delta\right)-\delta e^{-kt_E}\right]\right\}^m\approx\exp\left(-\eta\left[\beta-\delta\left(1-e^{-kt_E}\right)/t_E\right]\cdot t\right)$$

where $\eta=t_E/\left(t_E+t_D\right)$ is the scanning duty cycle.

By splitting one t_E into n pieces of t_E/n, the fluorescence gain is

$$\rho(n;\delta)=\frac{\left[R_{\text{cont}}\left(t_E\ \ n\right)\right]^n}{R_{\text{cont}}\left(t_E\right)}=\frac{\left[1+\delta-\delta\exp\left(-kt_E/n\right)\right]^n}{1+\delta-\delta\exp\left(-kt_E\right)}$$

Photobleaching from the first triplet state (T_1) is not significant

If photobleaching from the first triplet-state (T_1) dominated, it would primarily occur during t_D. The triplet-state accumulation in t_E would be

$$P_T\left(t_E\right)=P_0\frac{\varepsilon k_{ISC}}{k}e^{-\beta t_E}\left(1-e^{-kt_E}\right)$$

With long enough t_D, the photobleached population from T_1 in each scan would be

$$B_{T1}\left(t_E\right)=\frac{k_{bT1}}{k_{bT1}+\tau_T^{-1}}P_T\left(t_E\right)$$

Therefore, the ratio of the photobleached population between with n pieces of separated exposure t_E/n and one single continuous exposure t_E is

$$r\left(n\right)\approx\frac{n\cdot B_{T1}\left(t_E/n\right)}{B_{T1}\left(t_E\right)}\approx\frac{n\left(1-e^{-kt_E/n}\right)}{1-e^{-kt_E}}$$

Note that $r\left(n\right)$ is always greater than one. Therefore, if photobleaching from T_1 dominated, faster scanning speed would cause more severe photobleaching, which contradicts with the experiments.

Sample preparation and labeling

The rat/mouse heart section samples were fixed in 10% formalin. The cell samples (Hela cells or isolated cardiomyocytes) were fixed with 4% paraformaldehyde. The heart sections were ~4 μm thick, embedded in paraffin following standard procedures. The sections were then mounted on slides and heated in an oven at 58°C for 2 hours. After heating, the section samples were deparaffinized with 100% xylene for 3 × 5 minutes with agitation, placed in decreasing concentrations of ethanol (100%, 95%, 85% and 70%) for 2 × 5 minutes, and finally equilibrated in PBS. To achieve antigen unmasking, the heart section samples were immersed in a citrate-based antigen-unmasking solution (Vector Labs, catalog #H-3300; 3.75 ml in 400 ml distilled H_2O), microwaved using 7 cycles (2 minutes each) of high-heat, with 1 minute cooling between consecutive heating cycles. After antigen unmasking (heart sections) or fixation (cells), samples were washed in PBS 3 × 5 minutes, equilibrated in 5% NGS-1% BSA-PBS for 30 minutes at room temperature (RT) to block nonspecific labeling, and then incubated with primary antibodies at appropriate dilutions in 0.5% NGS-0.1% BSA-PBS overnight at 4 °C in a humidity chamber. The samples were then rinsed with PBS 3 × 5 minutes. The heart section samples were equilibrated to block again for 1 hour at RT. The samples were then incubated with the secondary antibody in 0.5% NGS-0.1% BSA-PBS for 60 minutes at RT. After washed 3 × 5 minutes in PBS, the samples were finally mounted using ProLong® Gold (Life Technologies, USA) for imaging. Blocking and antibody dilution buffers for the cell samples contained 0.5% Triton X-100 for permeabilization.

Supplementary table

Supplementary Table S1. Information of the biological samples

Sample	Dye	Cell/tissue type	Primary antibody	Excitation laser	Depletion laser
#1	Atto 647N	Hela cell	Anti-ADP/ATP carrier	635 nm, ~150 ps pulses, repetition rate 80 MHz	750 nm, ~400 ps pulses, repetition rate 80 MHz
#2	Atto 647N	Rat heart tissue section	Anti-Cytochrome c	Same as sample #1	Same as sample #1
#3	Oregon green 488	Mouse tissue section	Anti-VDAC	485 nm, CW	592 nm, CW
#4	Abberior STAR 635P	Isolated mouse cardiomyocyte	Anti-Cav1.2	Same as sample #1	Same as sample #1
#5	Alexa Fluor 647	Isolated mouse cardiomyocyte	Anti-Cav1.2	Same as sample #1	Same as sample #1
#6	Chromeo 494	Isolated mouse cardiomyocyte	Anti-Cav1.2	485 nm, ~150 ps pulses, repetition rate 80 MHz	Same as sample #1
#7	Alexa Fluor 488	Mouse tissue section	Anti-VDAC	485 nm, CW	592 nm, CW

Supplementary figures

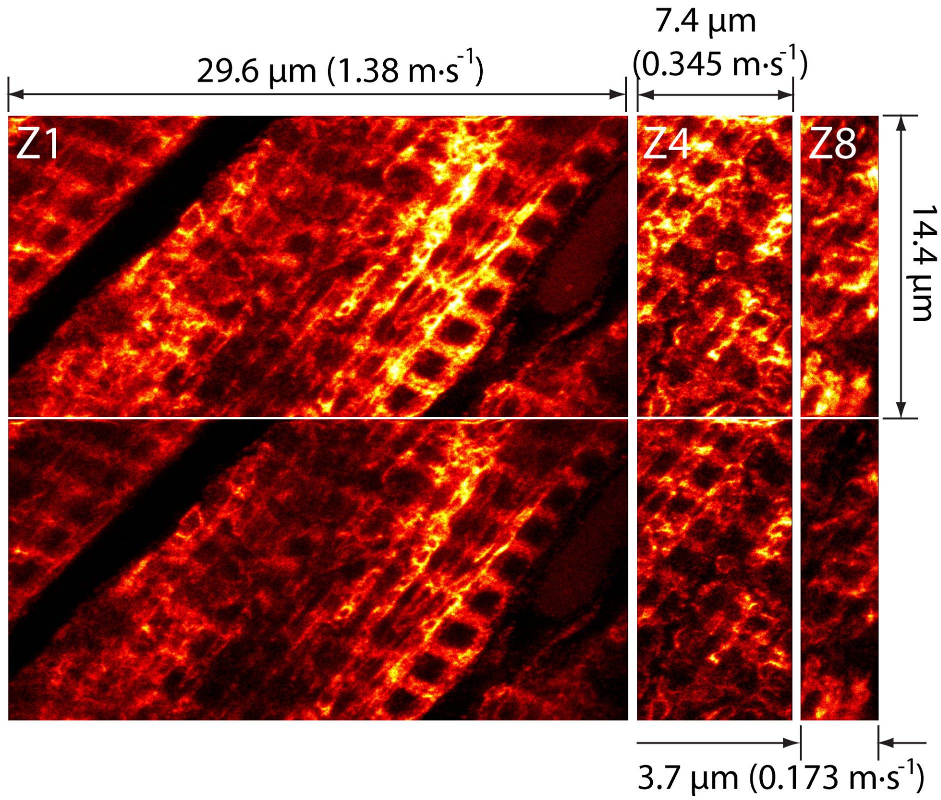

Supplementary Fig S1. Images taken at Zoom 1 (Z1), Zoom 4 (Z4) and Zoom 8 (Z8) for Sample #2. At all zooms, images have a pixel size of 15 × 15 nm. Images were cropped to only keep the portion with ≥90% of the maximum scanning speed. At Z1, the image width is 29.6 µm (1976 pixels). At Z4, the width is 7.4 µm (494 pixels), 1/4 of Z1. At Z8, the width is 7.4 µm (247 pixels), 1/8 of Z1. All zooms have the same height (14.4 µm; 960 pixels). Because of the fixed scanner frequency, the scanning speed at Z1 (1.38 m·s^{-1}) is 4 times as fast as Z4 (0.345 ms $^{-1}$), and 8 times as fast as Z8 (0.173 ms $^{-1}$). The upper panels show images taken in 3 minutes of normalized imaging time, and the lower panels show images taken in the next 3 minutes. Image intensity decay due to photobleaching is 69% (Z1), 63% (Z4), and 53% (Z8), respectively.

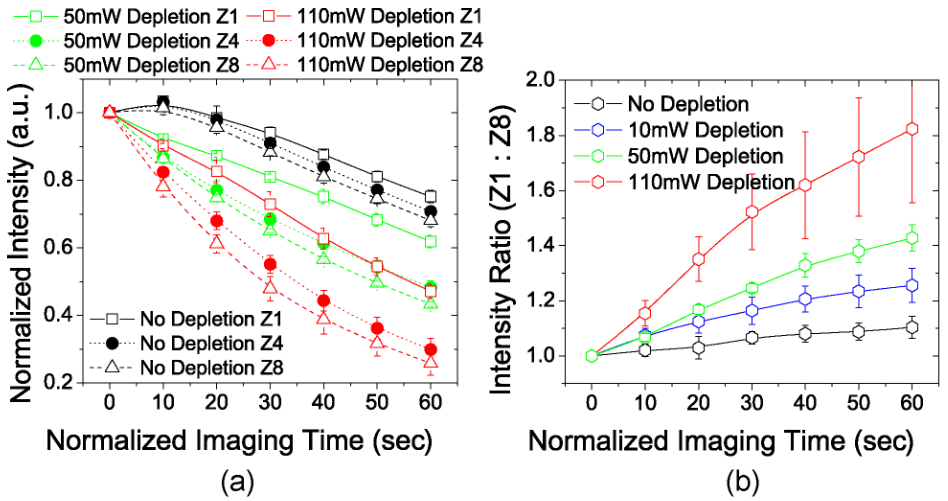

Supplementary Fig S2. Photobleaching rates in Sample #2 with varying depletion laser power. (a) Normalized image intensity decay due to photobleaching as a function of illumination (measured by the normalized imaging time). Higher scanning speed (lower zoom) results in slower decay. (b) Intensity ratio between Zoom 1 and Zoom 8 is greater than one, and increases with time and growing depletion laser power. All lines are a guide for the eye.

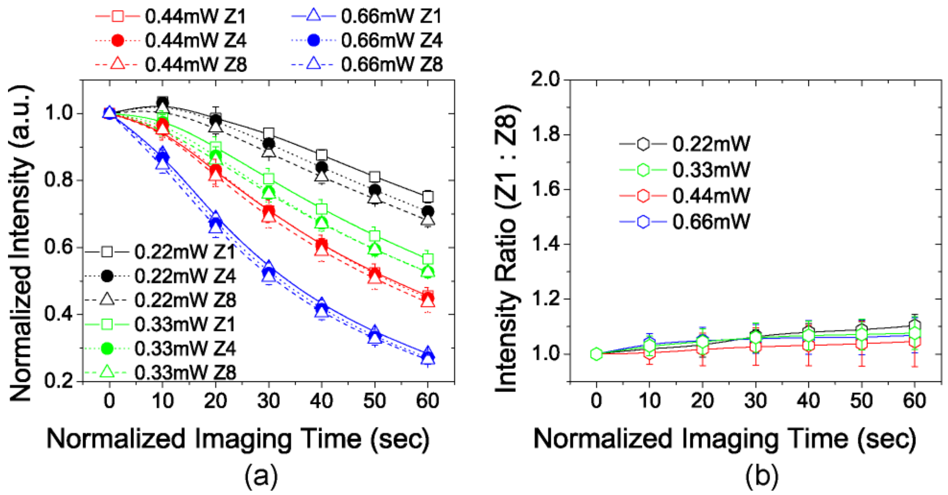

Supplementary Fig. S3. Photobleaching in Sample #2 without depletion (i.e., in regular confocal condition). (a) Image intensity decay curves. (b) Intensity ratio between Zoom 1 and Zoom 8 as function of illumination dose. Normalized image intensity is similar at all 3 zooms. Difference is at most ~15%. All lines are a guide for the eye.

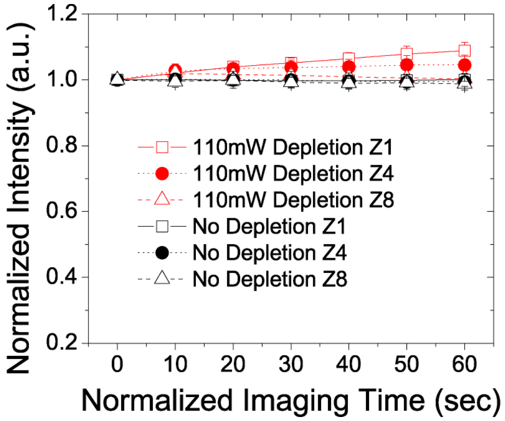

Supplementary Fig S4. Photobleaching caused by depletion laser only (red curves) in Sample #2. Image intensity slightly increased with time, meaning that the depletion laser alone do not cause photobleaching. Black curves represent background photobleaching caused by a low power (16 μW) excitation laser, which is almost zero. Lines are a guide for the eye.

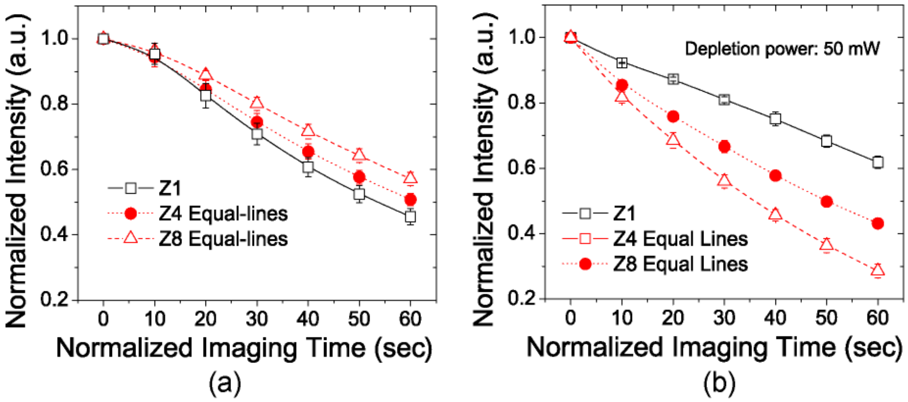

Supplementary Fig S5. Photobleaching under the equal-lines conditions for Sample #2. Zoom 1 data (black) is used as the reference. Red data points were taken under the equal-lines condition, using lower excitation irradiance. (a) In regular confocal condition, the equal-lines condition causes slower bleaching because lower laser power reduces nonlinear photobleaching. (b) In STED microscopy, the depletion power had to be kept constant (50 mW) to maintain resolution, and the equal-lines condition causes faster photobleaching because of extra depletion illumination. All lines are a guide for the eye.

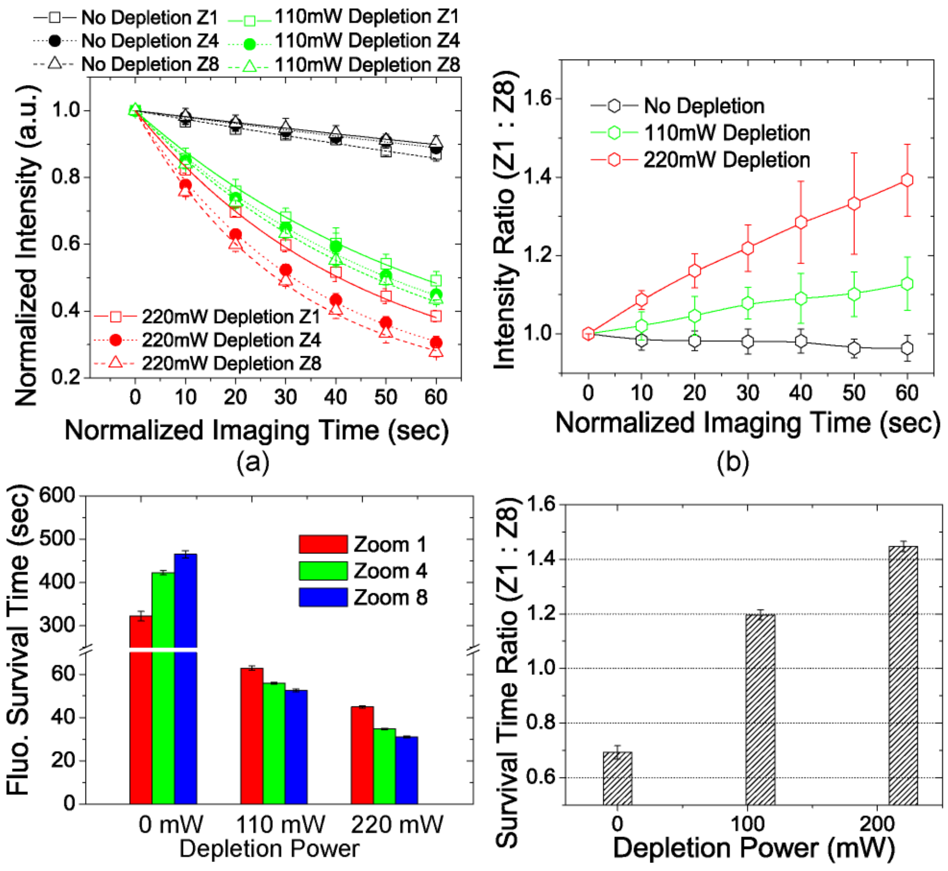

Supplementary Fig.S6. Photobleaching rates in Sample #3 with varying depletion laser power. (a) Image intensity decay data points were fitted to Eq. (3). (b) Intensity ratio between Zoom 1 and Zoom 8 increases with time and growing depletion laser power. Lines are a guide for the eye. (c) Fluorophore survival time with different depletion laser power at different zooms. Lower zooms (faster scanning speed) have longer survival time when depletion is not zero. (d) Ratio of the survival time between Zoom 1 and Zoom 8 is greater than one and increases with growing depletion power.

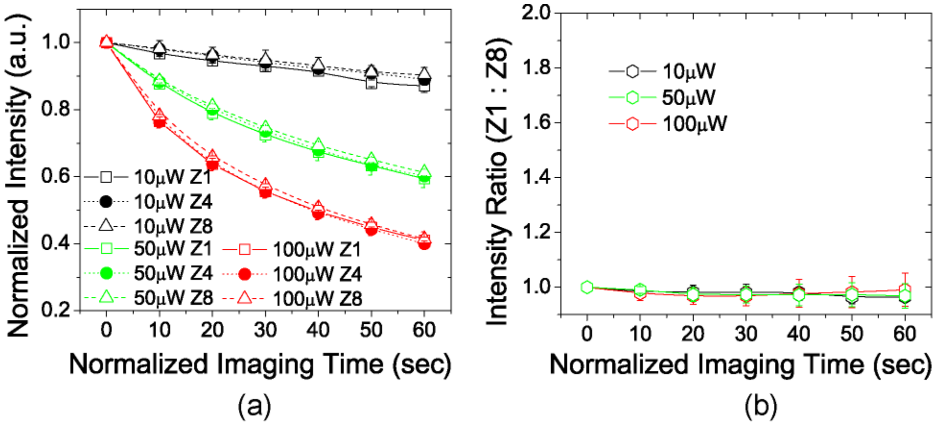

Supplementary Fig.S7. Photobleaching in Sample #3 under regular confocal condition (zero depletion). (a) Image intensity decay curves. (b) Intensity ratio between Zoom 1 and Zoom 8 as function of time. At high excitation power photobleaching rate is comparable to that in STED microscopy, but photobleaching rates at different zooms are almost the same. All lines are a guide for the eye.

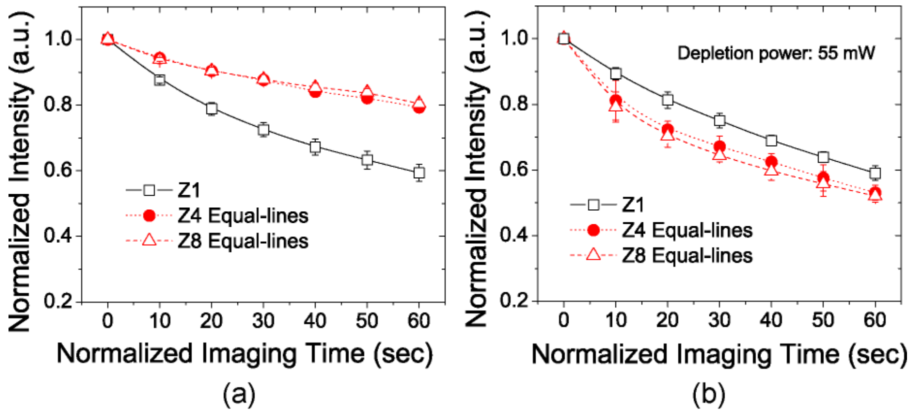

Supplementary Fig S8. Photobleaching under the equal-lines conditions for Sample #3. Zoom 1 data (black) is used as the reference. Red data points were taken under the equal-lines condition, using lower excitation irradiance. (a) In regular confocal condition, the equal-lines condition causes slower bleaching because lower laser power reduces nonlinear photobleaching. (b) In STED microscopy, the depletion power had to be kept constant (55 mW) to maintain resolution, and the equal-lines condition causes faster photobleaching because of extra depletion illumination. All lines are a guide for the eye.

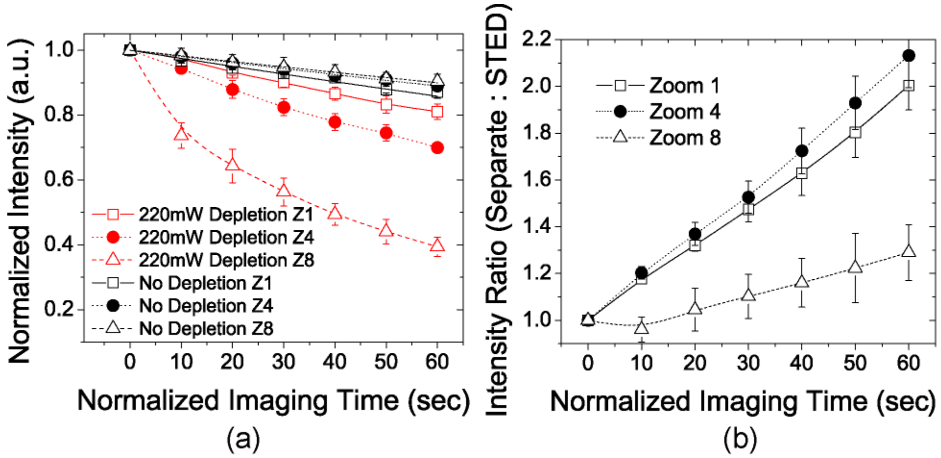

Supplementary Fig S9. (a) Image intensity decay with separated excitation and depletion illumination in Sample #3. The red data points use both excitation laser beam (485 nm, 50 μW) and depletion laser beam (592 nm, 220 mW), but they were never switched on at same time. Black curves are with excitation laser on only. Significant photobleaching was caused by depletion laser alone. At higher zoom (slower scanning speed), depletion-only photobleaching is more severe. (b) Photobleaching caused by separated excitation and depletion illumination is slower than STED photobleaching. The image intensity ratio between separate illumination and STED is almost always greater than one and keeps increasing with time. All lines are a guide for the eye.

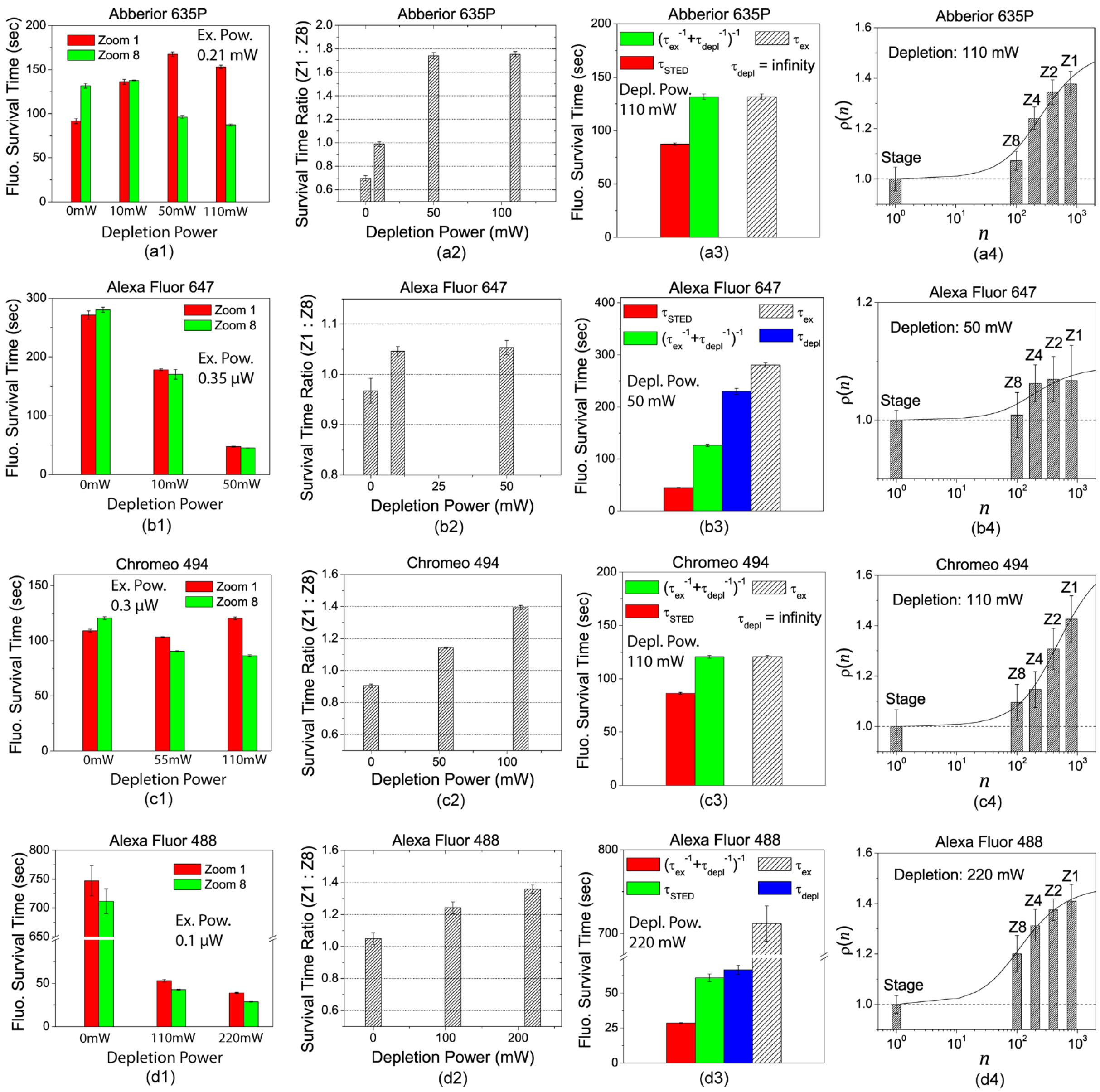

Supplementary Fig S10. Photobleaching of four fluorescent dyes (a: Abberior STAR 635P; b: Alexa Fluor 647; c: Chromeo 494; d: Alexa Fluor 488) in STED microscopy. a1—d1 show the fluorophore survival time at Zoom 1 and Zoom 8 with varying depletion power. a2—d2 show the ratio of the fluorophore survival time between Zoom 1 and Zoom 8 as a function of depletion power. a3—d3 compare the fluorophore survival time under four conditions: excitation-only (τ_{ex}); depletion-only (τ_{depl}); with the excitation light and the depletion light illuminating separately ($\left(\tau_{ex}^{-1}+\tau_{depl}^{-1}\right)^{-1}$); and in STED imaging ($\tau_{\mathrm{STED}}$). a4—d4 show the fluorescence gain ratio as a function of the exposure divisor (similar to Fig. 9B—9D) and the data fitting (solid line) to Eq. (4). The values of the fitted parameters are summarized in Table 2.

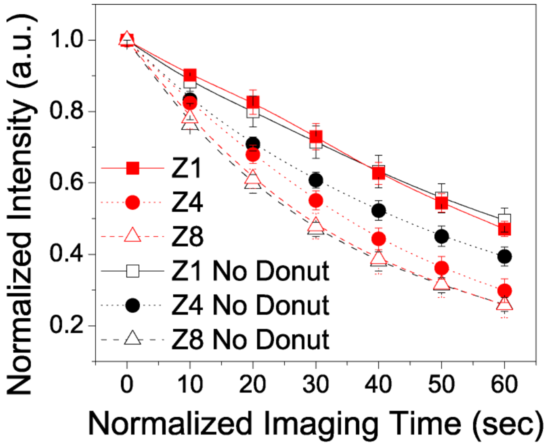

Supplementary Fig S11. Image intensity decay with 0.22 mW excitation power and 110 mW depletion laser power in Sample #2. The red curves show results with a hollow depletion laser beam, whereas the black curves are results with a Gaussian depletion beam. The difference is small, especially at Zoom 1 and Zoom 8.

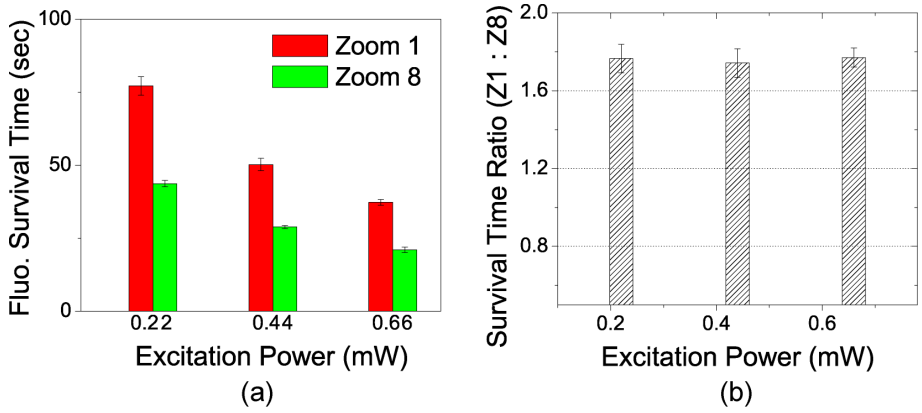

Supplementary Fig S12. Impact of excitation laser power on the fluorophore survival time of Atto 647N. (a) Fluorophore survival time of Atto 647N at Zoom 1 and Zoom 8 with various excitation power (0.22 mW, 0.44 mW, and 0.66 mW). The depletion power was fixed at 110 mW. (b) The ratio of the survival time between Zoom 1 and Zoom 8 barely changes with varying excitation power.
